# Supplementary material for: Linear dicentric chromosomes in bacterial natural isolates reveal common constraints for replicon fusion
Source: mBio. 2025 May 20;16(6):e01046-25. doi: 10.1128/mbio.01046-25 (PMC12153318; doi:10.1128/mbio.01046-25)
Supplement: Supplemental Material — Supplemental figures and tables. [file mbio.01046-25-s0001.pdf]

Supplementary Information for

Linear dicentric chromosomes in bacterial natural isolates reveal common constraints for replicon fusion

Ram Sanath-Kumar, Arafat Rahman, Zhongqing Ren, Ian P. Reynolds, Lauren Augusta, Clay Fuqua, Alexandra J. Weisberg, Xindan Wang

Correspondence:

Alexandra J. Weisberg
Email: alexandra.weisberg@oregonstate.edu

Xindan Wang
Email: xindan@iu.edu

This PDF file includes:

Figures S1 to S2
Tables S1 to S3
SI References

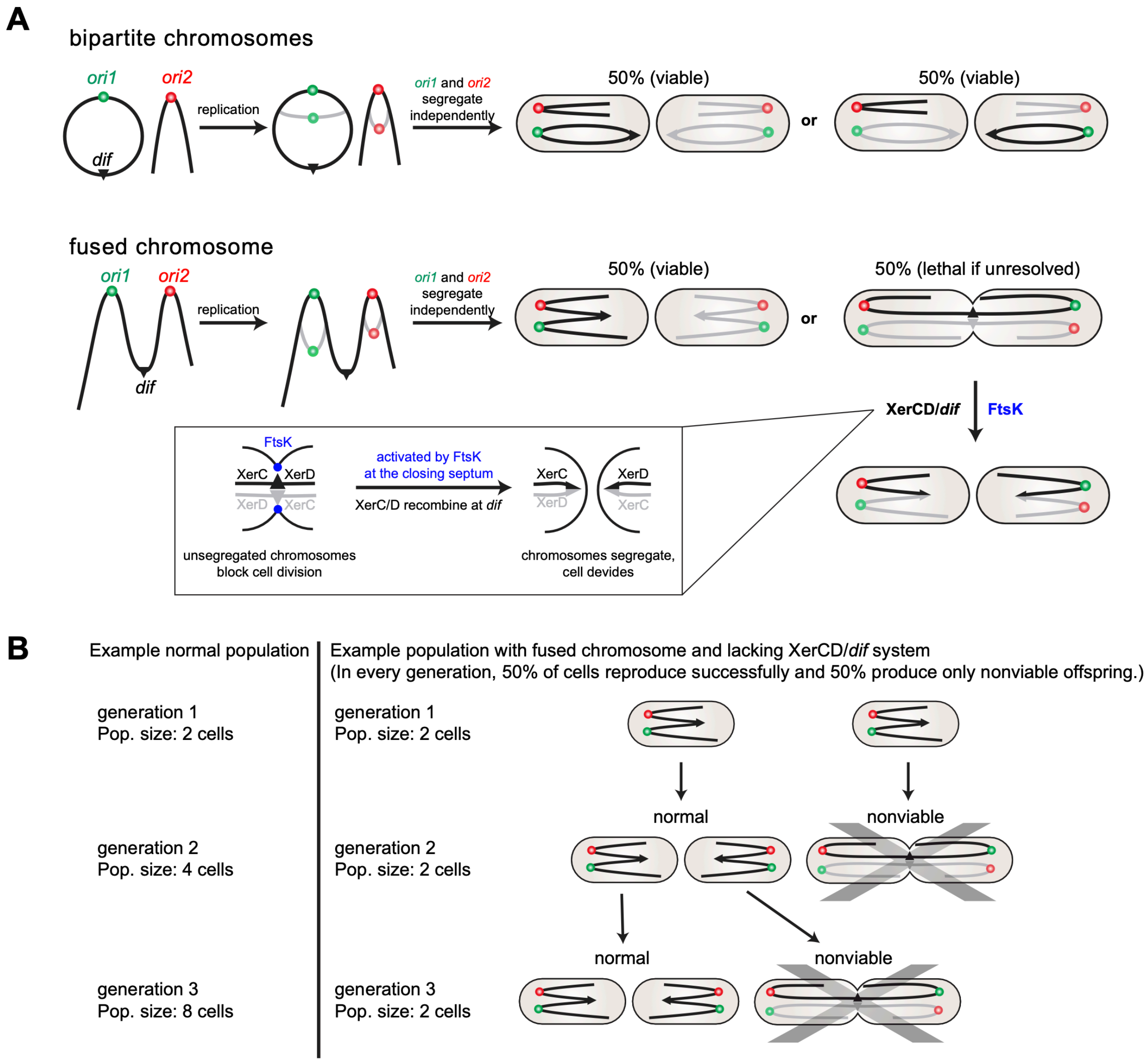

Figure S1. Fusion chromosomes require the FtsK-XerCD-*dif* system for survival. (A) Schematics models adapted from (1). After replication initiation, bipartite chromosomes separate independently and randomly into daughter cells. Both combinations will result in viable cells. For the fused chromosome, random segregation of *ori1* and *ori2* will generate 50% of cells with intact fusion chromosomes in each daughter cell, resulting in viable progeny. However, in 50% of the cases in every generation, the copies of *ori1* and *ori2* that belong to the same DNA molecule translocate to opposite daughter cells. If the situation is unresolved, the chromosomes will block cell division and cause cell death. Stimulated by FtsK at the closing septum, XerCD will recombine at the *dif* sites to resolve the unsegregated chromosomes. **(B)** Using an example population of two cells to calculate the population size through three generations. Left panel, normal population. Right panel, cells with a fused chromosome but lacking the XerCD/*dif* system. In these cells, in every generation, 50% of cells will reproduce successfully and 50% will produce only nonviable offspring. Through generations, the population size will not grow.

47-2

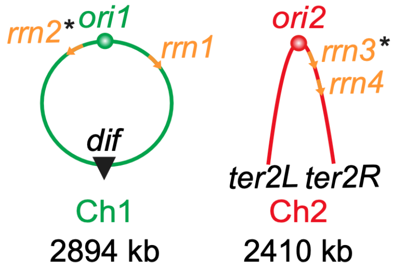

Figure S2. Locations of *rrn* operons on the chromosomes of strain 47-2. Asterisks mark the predicted sites of chromosome fusion in CFBP_2407 and CFBP_2642.

Table S1. Bacterial strains used in this study.

Strain	Reference	Figure
A. tumefaciens 47-2 (BV1 biovar; env. isolated binary chromosome)	This Study	1, 2A, 3, 4A, 5
A. tumefaciens CFBP_2407 (BV1 biovar; env. isolate fused chromosome)	This Study	1, 2B, 3, 4A, 5
A. tumefaciens CFBP_2642 (BV1 biovar; env. isolate fused chromosome)	This Study	1, 2C, 3, 4A, 5

Table S2. Nanopore sequencing samples generated in this study.

Sample name	Figure	Reference	Identifier
47-2	1AB	This Study	SRR31799202
CFBP_2407	1AB	This Study	SRR31799201
CFBP_2642	1AB	This Study	SRR31799200

Table S3. Hi-C and WGS samples generated in this study.

Sample name	Figure	Reference	Identifier
HiC_47-2_rep1	1C, 2B, S1A	This Study	GSM8696812
HiC_2407_rep1	1D, 2B, S1A	This Study	GSM8696813
HiC_2642_rep1	1E, 2B, S1A	This Study	GSM8696814
WGS_47-2_rep1	1ABC, 2B. S1B	This Study	GSM8696815
WGS_2407_rep1	1ABD, 2B, S1B, S2A	This Study	GSM8696816
WGS_2642_rep1	1ABE, 2B. S1B, S2A	This Study	GSM8696817

SI References

1. Q. Liao, Z. Ren, E. E. Wiesler, C. Fuqua, X. Wang, A dicentric bacterial chromosome requires XerC/D site-specific recombinases for resolution. *Curr Biol* **32**, 3609-3618 e3607 (2022).
